# Induction of Membrane Ceramides: A Novel Strategy to Interfere with T Lymphocyte Cytoskeletal Reorganisation in Viral Immunosuppression

**DOI:** 10.1371/journal.ppat.1000623

**Published:** 2009-10-16

**Authors:** Evelyn Gassert, Elita Avota, Harry Harms, Georg Krohne, Erich Gulbins, Sibylle Schneider-Schaulies

**Affiliations:** 1 Institute for Virology and Immunobiology, University of Würzburg, Würzburg, Germany; 2 Department of Electron Microscopy, University of Würzburg, Würzburg, Germany; 3 Department for Molecular Biology, University of Essen, Essen, Germany; University of Southern California School of Medicine, United States of America

## Abstract

Silencing of T cell activation and function is a highly efficient strategy of immunosuppression induced by pathogens. By promoting formation of membrane microdomains essential for clustering of receptors and signalling platforms in the plasma membrane, ceramides accumulating as a result of membrane sphingomyelin breakdown are not only essential for assembly of signalling complexes and pathogen entry, but also act as signalling modulators, e. g. by regulating relay of phosphatidyl-inositol-3-kinase (PI3K) signalling. Their role in T lymphocyte functions has not been addressed as yet. We now show that measles virus (MV), which interacts with the surface of T cells and thereby efficiently interferes with stimulated dynamic reorganisation of their actin cytoskeleton, causes ceramide accumulation in human T cells in a neutral (NSM) and acid (ASM) sphingomyelinase–dependent manner. Ceramides induced by MV, but also bacterial sphingomyelinase, efficiently interfered with formation of membrane protrusions and T cell spreading and front/rear polarisation in response to β1 integrin ligation or αCD3/CD28 activation, and this was rescued upon pharmacological or genetic ablation of ASM/NSM activity. Moreover, membrane ceramide accumulation downmodulated chemokine-induced T cell motility on fibronectin. Altogether, these findings highlight an as yet unrecognised concept of pathogens able to cause membrane ceramide accumulation to target essential processes in T cell activation and function by preventing stimulated actin cytoskeletal dynamics.

## Author Summary

The ability of measles virus (MV) to impair T cell–dependent immune responses noted more than 100 years ago continues to be central to the severe generalised immunosuppression by this virus. Much has been learned about receptors and mechanisms, which determine the predilection of MV for hematopoetic cells. In contrast, little is known on a molecular level how MV interferes with processes relaying extracellular signals to T cells which translate into reorganisation of their cytoskeleton as required for their migration and cell–cell communication. Our study now shows that MV activates sphingomyelinases and ceramide accumulation in T cell membranes and this severely impairs integrity and stimulated reorganisation of their actin cytoskeleton, morphologically resulting in collapse of actin based protrusions, and functionally in impaired motility. During these studies, we appreciated, however, that cues other than MV eliciting ceramide accumulation in general also caused T cell paralysis. This indicates that ceramide accumulation and its consequences are not only a novel concept for MV-induced T cell silencing, but rather reflects a general strategy which may apply to extracellular ligands including pathogens able to promote plasma membrane ceramide accumulation, thereby preventing T cell activation.

## Introduction

Activation of sphingomyelinases (SMases) which differ in their respective pH optimum and activity largely accounts for accumulation of membrane ceramides; that of the acid sphingomyelinase (ASM) was related to the transformation of small membrane cholesterol and sphingomyelin (SM) enriched microdomains (also referred to as rafts) into large, ceramide-enriched membrane platforms in response to a variety of external stimuli [1],[2]. In hematopoetic cells, these include ligation of death receptors [3],[4], CD40, FcγRII and CD28 [5]. Certain pathogens such as *P. aeruginosa*, *S. aureus* and rhinovirus also promote and rely on formation of ceramide-enriched platforms for entry (reviewed in [6],[7]), while these interfere with fusion-dependent uptake of HIV [8]–[10]. Regulation of lateral diffusion and recruitment of surface receptors and membrane-proximal signalling complexes are general mechanisms of ceramide-enriched platforms to enhance initiation of or modulate signalling pathways [2], [11]–[13], including integrin-signalling in endothelial and neural cells [14],[15], and cytoskeletal remodeling in breast cancer MCF-7 cells independently of apoptotic ceramide signalling [16],[17].

For migratory cells such as T cells, dynamic reorganisation of the actin cytoskeleton in response to external soluble and cell-associated stimuli are required for polarisation, motility, scanning of the APC surface, and lastly, formation of immunological synapses (IS) [18]–[20]. Regulated sorting and clustering of receptors to cellular subdomains such as the leading edge or uropod during migration, or the IS and the distal pole, respectively, relies on a dynamic coupling of surface receptors to the actin cytoskeleton by linker proteins including ezrin/radixin/moesin (ERM) family proteins or talin. Morphologically, retraction of actin-based protrusions facilitates the formation of interaction platforms and exposure of surface molecules from crypts [18]. Given the importance of these processes, their deregulations by viruses expectedly would be of great relevance to immunomodulation, in particular, immunosuppression.

Measles virus (MV) was the first pathogen recognised as immunosuppressive. Its ability to prevent entry of stimulated T lymphocytes into the G_1_ phase of the cell cycle *in vitro*, is reflected by the general impairment of T lymphocytes isolated from patients to expand in response to mitogenic activation [21],[22]. Since within this population only a minor fraction of T cells is actually infected [23], T cell silencing is thought to occur independently of infection and rather predominantly result from signalling by the viral glycoprotein complex expressed on the surface of infected dendritic cells (DCs) [22],[24]. Surface bound MV colocalised with GM1 on primary T cells, indicating that MV signalling is initiated within detergent resistent membrane microdomains, and this occurs independently of CD150, the MV uptake receptor [25]. MV surface signalling ablates activation of the phosphatidyl-inositol-3-(PI3)/Akt kinase pathway *in vitro* and *in vivo* [26]–[28] and consequently, T cell activation, adherence, polarisation and lamellopodia formation in response to TCR ligation or integrin signalling as well as receptor clustering in conjugates formed with mature DCs are impaired [29]. In the absence of external activation, MV signalling to primary human T cells was found to cause inhibition of Akt kinase activation, dephosphorylation of ERM proteins, activation of RhoA and collapse of actin-based microvilli [29] all of which are consistent with activation of SMases and/or membrane ceramide accumulation in non-hematopoetic cells [16], [17], [30]–[33].

We now show that MV signalling causes activation of both NSM and ASM and ceramide accumulation on human T cells. Ablation of ASM or NSM protein expression or function did not rescue MV induced-induced inhibition of T cell proliferation yet effectively abolished interference with actin cytoskeletal dynamics. In line with these observations, T cell exposure to MV or bacterial SMase (bSMase) also modulated T cell motility, altogether suggesting a novel function for membrane ceramide in regulating T cell polarisation and motility, and thereby T cell dynamics.

## Results

### MV causes ASM and NSM activation and accumulation of membrane ceramides on T cells

**Figure 1:**
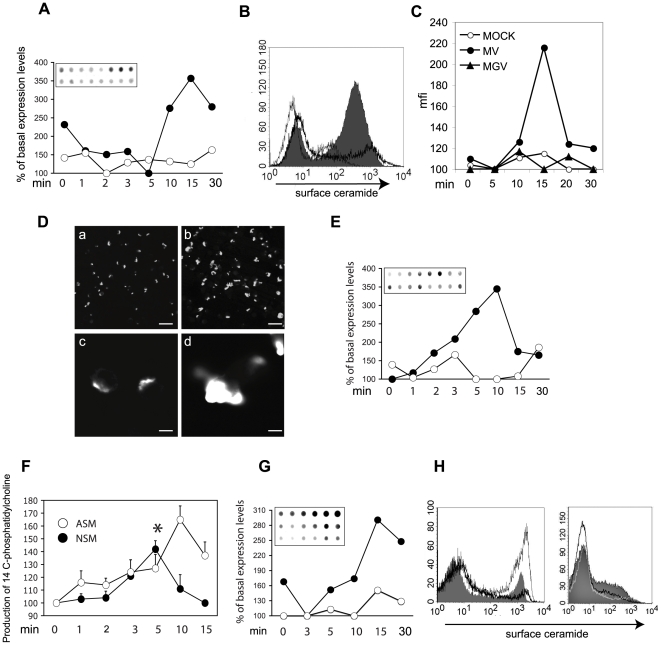
MV causes SMase activation and membrane ceramide accumulation in human T cells. (A–D) Extracellular oriented ceramide accumulation was detected on Jurkat T cells exposed to MV (black symbols) or a MOCK preparation (white symbols) after the time intervals indicated. (A) By spot assay. Levels indicated were normalised to those measured on untreated cells; one out of three independent experiments is shown (inset: MV: upper, MOCK bottom row). (B–D) By surface detection. (B,C) FACScan on cells exposed to (B) MOCK (solid black curve) or MV (filled histogram) (exemplified for a 10 min exposure) (the grey line indicates staining of the isotype control antibody) or (C) MOCK, MGV (black triangles) or MV for the time intervals indicated (min), (D) by immunofluorescent staining (exemplified for a 10 min exposure to MOCK (a and blow up in c) or MV (b and blow up in d) (bars represent 25 (a, b) or 5 µm (c, d)). (E) Surface display of ASM was determined on Jurkat T cells exposed to MOCK (white symbols) or MV (black symbols) for the time intervals indicated by spot assay (inset; MV upper, MOCK bottom row (as in (A), values were normalised to those measured on untreated cells)). (F) ASM (open symbols) and NSM (closed symbols) activation by MV was measured by conversion of ^14^C-sphingomyelin to ^14^C-phosphorylcholine by extracts prepared from MV or MOCK-treated cells within 1 hr. Values shown indicate production of ^14^C-phosphorylcholine by MV over time, each expressed in relation to the respective MOCK control; levels of NSM activation significantly exceeding the background (t-test, p = 0.01) are indicated by an asterisk (data were generated in three independent experiments, standard deviations are indicated). (G) ASM surface display on primary human T cells in response to MOCK (white symbols) or MV (black symbols) over time measured by spot assay (inset; MV middle, MOCK bottom row). CD28-activation was used as a positive control (inset, upper row). (H) Left panel: Primary human T cells were left untreated (grey curve) or pre-exposed to amitriptyline (filled histogram) or GW4869 (black curve) for 2 hrs prior to MV exposure for the time intervals indicated, stained for ceramide surface display and analysed by FACScan. Right panel: Primary T cells were transfected using a scrambled (filled histogram) or a NSM2-specific siRNA (black and white line) prior to exposure to MOCK (white line) or MV (filled histogram and black line).

**Figure 2:**
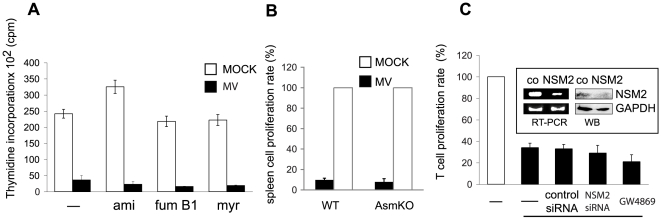
MV-induced SMase activation does not account for MV interference with stimulated T cell expansion. (A) Human primary T cells left untreated or treated with amitriptyline (ami), fumonisin B (Fum B1), myriocin (myr) for 2 hrs were exposed to MV or MOCK in the presence of FIP and αCD3/CD28 stimulated. Proliferation was monitored by ^3^H-thymidine incorporation during the last 16 hrs of culture. One representative out of three independent experiments (each performed in triplicates) using three different donors is shown. (B) Spleen cells isolated from AsmKO or wild-type mice were exposed to MOCK or MV and αCD3/CD28 stimulated. Proliferation rates were determined as described in A, with those seen for the MOCK controls set to 100%. (C) Human primary T cells were either left untreated, or transfected with a scrambled (control siRNA) or a NSM2-specific siRNA (NSM2) or pretreated with the NSM-inhibitor (GW4869). 24 hrs after inhibitor treatment or 96 hrs after transfection, cells were exposed to MV (black bars) or MOCK (white bar), αCD3/CD28-stimulated and proliferation rates were determined as described in (A). Values obtained (in three independent experiments) for MOCK were each set to 100% and those obtained MV exposed samples (black bottom line) were determined as relative to the individual MOCK controls. The efficiency of NSM2 silencing by control or the specific siRNA was controlled by RT-PCR and WB (inset).

To analyse the ability of MV to promote ceramide accumulation on the surface of T cells, Jurkat cells were exposed to MV or a MOCK preparation in the presence of a fusion inhibitory peptide. Subsequently, levels of cell surface ceramide were determined in an assay based on immunodetection of a ceramide-specific antibody bound to intact cells (spot assay) [34]. MV, but not MOCK or the fusion inhibitory peptide alone stimulated an approximately three-fold increase of extrafacial ceramide raising after 5 min and peaking at 15 min (normalised to basal expression levels determined for untreated cells) (Fig. 1A). In addition to this assay, also FACS analyses confirmed surface accumulation of ceramides on T cells in response to MV (Fig. 1B), but not to a recombinant expressing VSV G protein instead of the MV glycoproteins documenting their importance in surface ceramide induction (Fig. 1C). The appearance of extrafacial ceramide accumulations consistent with ceramide-enriched platforms was also detected by immunocytochemistry (exemplified for a 10 min MV exposure in Fig. 1D). To analyse whether MV induced surface ceramides relied on ASM activation, we analysed cell surface display of this enzyme by spot assay. Slightly preceding ceramides, extrafacial ASM started accumulating within 5 min after MV exposure, peaked at 10 min and subsequently declined (Fig. 1E). A biochemical assay using ^14^C-labeled sphingomyelin as a substrate, confirmed the kinetics of ASM activation by MV (Fig. 1F). Interestingly, transient activation of NSM significantly over background levels at 5 min post MV exposure was reproducibly detected (Fig. 1F). Importantly, MV caused an approximately threefold surface accumulation of ASM and ceramides also in primary human T cells though with a slightly slower kinetics than determined for Jurkat cells (Fig. 1G, and not shown). Pre-exposure to the ASM inhibitor amitriptyline efficiently prevented MV induced ceramide surface accumulation on Jurkat and primary T cells (Fig. 1H, left panel, and not shown). Interestingly, inhibition of NSM by a pharmacological (using GW4689 at concentrations not toxic to the target cells, Fig. 1H, left panel) or a genetic approach (using an NSM2-specific siRNA, the efficiency of which is exemplified in Fig. 2C) also prevented ceramide accumulation on primary or Jurkat T cells (Fig. 1H, right panel, and not shown), indicating that NSM activation by MV is essential for this phenomenon and may relate to that of ASM subsequently. Taken together, MV contact promotes activation of both ASM and NSM along with surface ceramide accumulation within few minutes on human T cells.

### Ceramide generation does not account for MV-induced inhibition of stimulated T cell expansion

If generation of membrane ceramides were to account directly for contact dependent MV inhibition of T cell proliferation, the latter should be abolished on ablation of SMase function or expression. As established, MV but not MOCK exposure prevented α-CD3/CD28 driven expansion of human T cells, and amitriptyline did not alter the effects of MV on T cell proliferation (Fig. 2A). MV-induced T cell inhibition was also insensitive to myriocin or fumonisin B1, indicating that this process did not involve *de novo* sphingolipid biosynthesis. Murine T cells also respond to MV signalling [26], and thus the impact of ASM activation on stimulated T cell expansion was assessed by analysing the sensitivity of α-CD3/CD28 driven proliferation of spleen cells isolated from Asm knockout (AsmKO) mice. These proved to be equally responsive to MV signalling as wild-type cells, indicating that Asm activation is not sufficient to cause MV proliferative arrest (Fig. 2B). Similarly, neither pharmacological nor genetic interference with NSM rescued MV-induced inhibition of αCD3/CD28-stimulated proliferation of human T cells (Fig. 2C). This was also true for cultures where both ASM and NSM activity had been inhibited and MV, but not exposure to C_16_ ceramide or bSMase efficiently inhibited α-CD3/CD28 stimulated T cell expansion as determined by a 16 hrs ^3^H-thymidine pulse after 48 hrs (not shown). Thus, ASM/NSM activation per se is not sufficient to cause MV T cell arrest.

### Ceramide generation accounts for MV-induced paralysis of microvillar membrane protrusions and T cell polarisation

**Figure 3:**
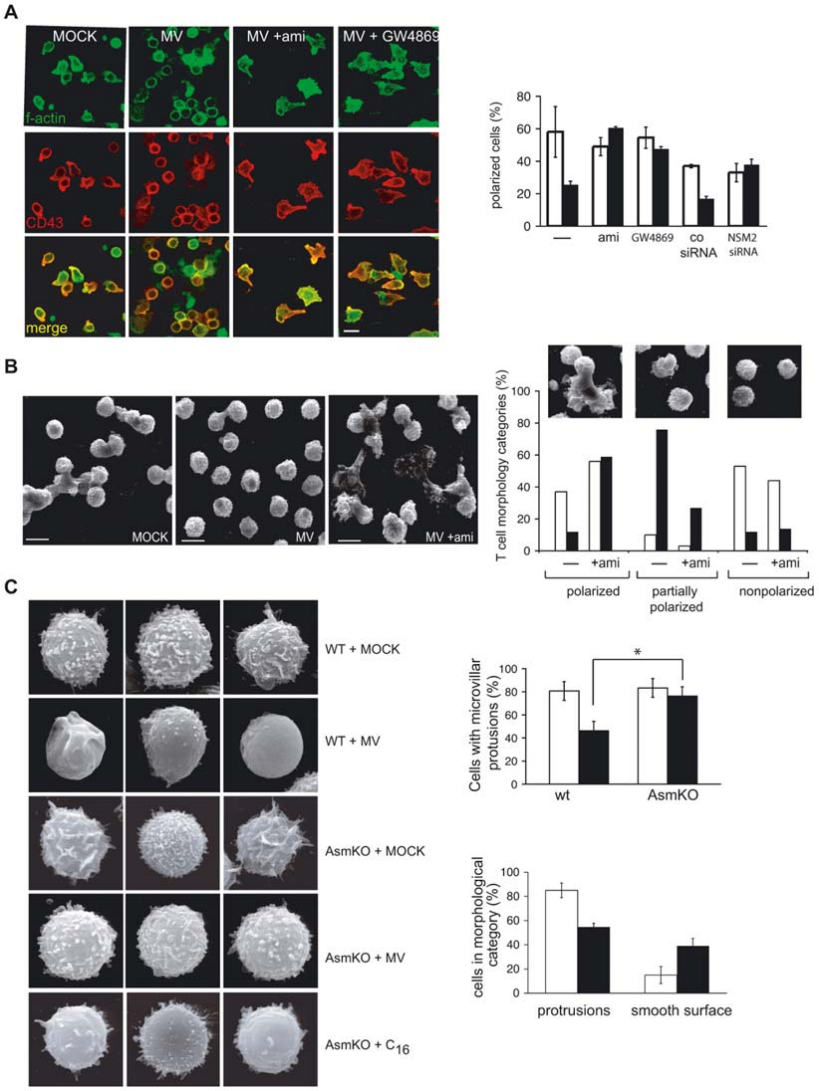
SMase activation accounts for MV-induced prevention of T cell polarisation and loss of membrane protrusions. (A) Human T cells were transfected with a NSM2-specific or a scrambled siRNA for 96 hrs or exposed to amitriptyline (ami) or GW4869 2 hrs prior to MOCK (right panel, white bars) or MV (right panel, black bars) exposure for 2 hrs at 4°C and subsequently seeded onto FN-coated slides. Cells were fixed after 30 min and stained for f-actin and CD43 (exemplified in the left panel, with the scale bar representing 5 µm) and the percentage of cells revealing a front-rear polarisation with CD43 enriched in the uropod was determined. At least 100 cells per culture were recruited into the analysis. (B) Human T cell cultures exposed to MOCK or MV (pre-exposed to amitriptyline for 24 hrs or not) were seeded onto FN coated slides and analysed by SEM (left panel; bars represent 5 µm). Right panel: Cells in each culture were scored into morphological categories (non-, partially or fully polarized; see insets for examples; 100 cells per culture were counted) and the respective percentage of cells exposed to MV (black bars) or MOCK (white bars) was determined. (C) Left panel: Spleen cells from wild-type (upper and second row) or AsmKO mice (middle to bottom row) were exposed to MOCK (upper and third row), MV (second and fourth row) or C_16_ ceramide (bottom row) prior to seeding onto FN and subsequent SEM analysis. Right, upper panel: Wild-type or AsmKO spleen cells exposed to MV (black bars) or MOCK (white bars) were seeded onto FN and the percentages of cells forming membrane protrusions in each culture were determined by SEM. Right, bottom panel: AsmKO cells exposed to MOCK (white bars) or C_16_ ceramide (black bars) were seeded onto FN and percentages of cells revealing membrane protrusions or with smoot appearance were determined. Both right panels: At least 100 cells were recruited into the analysis per culture. Levels of significance were determined using a t-test (p = 0.01).

We reasoned that ceramides could relate to the collapse of actin based microvillar structures on T cells exposed to MV associated with low phosphorylation levels of the ERM family cytoskeletal linker proteins ezrin and moesin [29]. Both features resembled findings reported for MCF-7 breast cancer cells, where cisplatin-induced ASM activation accounted for loss of lamellopodia/filopodia and p-ezrin [17]. In line with our previous observations, a substantial fraction of MV exposed human T cells remained small and rounded on fibronectin (FN) with condensed cortical actin instead of spreading or aquiring a front-rear polarity with enrichment of CD43 in the uropod ([29], and Fig. 3A). Strikingly, pre-exposure to amitriptyline, but also to GW4869 as well as siRNA-mediated NSM knock-down efficiently rescued the morphotrophic effects of MV on these cells (Fig. 3A) indicating that ceramides generated due to SMase activation essentially account for the loss of T cell polarisation. As revealed by scanning electron microscopy (SEM) analyses, microvillar structures projected from the majority of control T cells about 40% of which revealed a clearly polarised morphology, while in cultures exposed to MV, the majority of cells was devoid of organised surface projections and the fraction of polarised cells was substantially lower (Fig. 3B). In line with our confocal microscopical findings, amitriptyline pre-treated T cells exposed to MV polarised as efficiently as the controls and retained organised microvillar structures. Amitriptyline did not affect the morphology of partially and non-polarised cells present in either culture at roughly the same frequency (Fig. 3B). To prove the importance of SMase activation in this process directly, the morphology of spleen cells isolated from AsmKO mice on FN was analysed by SEM. Though membrane protrusions in murine spleen cells were overall shorter than those seen on human T cells, MV exposure caused a detectable loss of these structures in spleen cells of wild-tpye animals (Fig. 3C, left panel, first and second row). In contrast, AmsKO spleen cells, some of which spontaneously formed extended ruffles on FN (Fig. 3C, left panel, middle row), were refractory to MV-induced cytoskeletal collapse (Fig. 3C, fourth row and quantified in C, right upper panel), however, were highly susceptible to that induced upon exogenous membrane ceramide accumulation by exposure to C_16_-ceramide or bSMase (Fig. 3C, right bottom panel, and not shown). Altogether, these data indicate that MV interference with cytoskeletal dynamics in T cells, as reflected by the loss of membrane protrusions and responsivenenss to β1 integrin stimulated polarisation, relies on membrane ceramide generation.

**Figure 4 ppat-1000623-g004:**
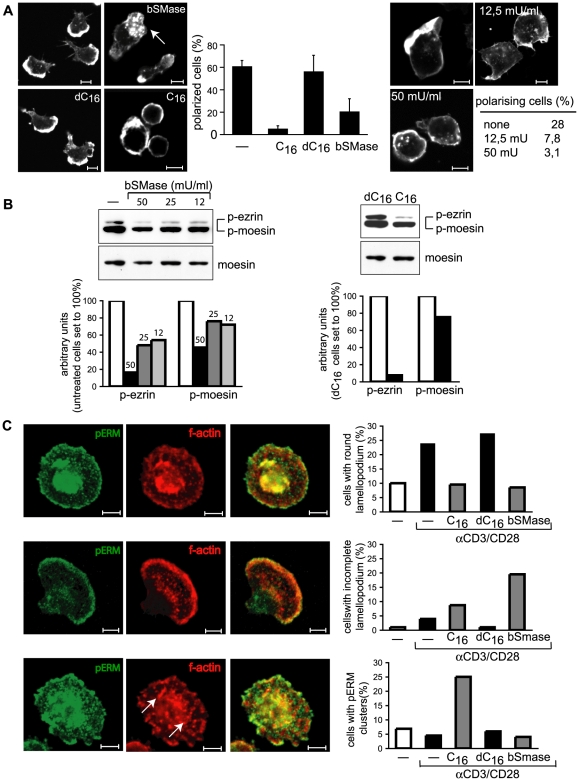
Ceramide generation abolishes T cell polarisation and spreading as well as pERM levels. Human T cells (left untreated or exposed to bSMase, C_16_ or dC_16_ were seeded onto (A) FN (left and middle panel; right panel shows Jurkat cells 30 min after FN seeding) or (C) αCD3/CD28 coated slides, and stained, in (A), for f-actin alone or in (C), together with pERM protein. F-actin aggregates consistent with formation of podosome-like structures are exemplified by arrows. The percentages of cells acquiring a polarised phenotype in (A) (middle and right panel) or in (C), differing in morphology by developing organised round or incomplete lamellopodia or revealing pERM clusters and podosome-like structures (arrows) are indicated (at least each 100 cells were counted per culture). Scale bars represent 5 µm. (B) Levels of p-ezrin/moesin were determined in primary human T cells left untreated or exposed to bSMase at the concentrations indicated (left panel) or exposed to dC_16_ or C_16_ (50 µm) (right panel), each for 2 hrs by WB. p-ezrin or p-moesin levels were quantified by densitometry (bottom graphs). Detection of moesin served as loading control.

To lend further proof to the hypothesis that membrane ceramide accumulation per se impedes stimulated T cell cytoskeletal dynamics, the impact of bSMase or addition of C_16_-ceramide (or inactive dihydro-C_16_ (dC_16_)) on β1 integrin stimulated T cell polarisation was analysed. When used at concentrations not interfering with T cell viability, bSMase and C_16_, yet not dC_16_ exposed T cells inefficiently spreaded and polarised on FN as revealed by a low frequency of cells acquiring the typical hand-mirror morphology (Fig. 4A, left and middle panel). Rather, actin-rich more central clusters with podosomal morphology were observed in a substantial percentage of cells, and this was particularly seen upon bSMase exposure (Fig. 4A, left panel, arrow). Though less efficiently than in primary T cells, FN seeding induced polarisation of cortical actin in a substantial percentage of Jurkat T cells, and this was dose dependently abrogated upon pre-exposure to bSMase or C_16_ (Fig. 4A, right panel, and not shown).

Since we have previously shown that ezrin/moesin phosphorylation is downmodulated in human T cells in response to MV [29], we analysed phosphorylation levels of these proteins (the ERM species expressed in human T cells [35]) in response to C_16_ and bSMase. Both compounds reduced p-ezrin levels, while p-moesin was less affected (Fig. 4B).

To analyse as to whether increase in membrane ceramides impairs the ability of T cells to organise actin cytoskeletal structures to stimuli other than FN, primary T cells were exposed to bSMase or C_16_ (or dC_16_) and seeded onto costimulatory slides coated with α-CD3/CD28 antibodies (Fig. 4C). About 25% of untreated or dC_16_ exposed cells efficiently spreaded to organise extended lamellopodia with pERM proteins lining and thereby tethering the plasma membrane to the underlying f-actin cytoskeleton (Fig. 4C, exemplified in the upper row and graph). In line with ceramides interfering with stimulated cytoskeletal dynamics, lamellopodia formation (as determined by f-actin detection) barely occurred in C_16_ or bSMase exposed cultures (Fig. 4C, upper graph). In those, costimulation induced morphological alterations were largely atypical with incomplete and asymmetrical lamellopodia (prevailing upon bSMase exposure) (exemplified in Fig. 4C, middle row and graph), or a limited spreading ability (mainly seen after C_16_ treatment) (Fig. 4C, bottom row and graph). Though C_16_ and bSMase caused overall loss of p-ezrin, p-moesin was much less affected (Fig. 4B). Thus, not surprisingly, p-ERMs recognised by the polyclonal antibody were readily detectable by immunofluorescence performed on T cells exposed to these compounds. Interestingly, in bSMase or C_16_-exposed cells, pERMs (including both ezrin and the more abundant moesin proteins) apparently revealed an aberrant subcellular distribution in response to TCR ligation. In bSMase treated cells, both f-actin and a very high proportion of pERMs localised to peripheral sites of the asymmetrical lamellopodia, yet revealed little overlap (Fig. 4C, middle row). In contrast, C_16_ promoted appearance of actin- and pERM-rich clusters consistent with attempted formation of podosomes which only partially overlapped and revealed a somewhat random distribution both at peripheral and internal sites (Fig. 4C, bottom panels). In summary, our results are consistent with membrane ceramides interfering with reorganisation of the actin cytoskeleton in response to both FN or TCR ligation in primary T cells and this is associated with a loss of predominantly p-ezrin, and this mirrors our observations made after MV exposure of these cells [29].

### MV-induced ceramide impairs T cell motility

**Figure 5:**
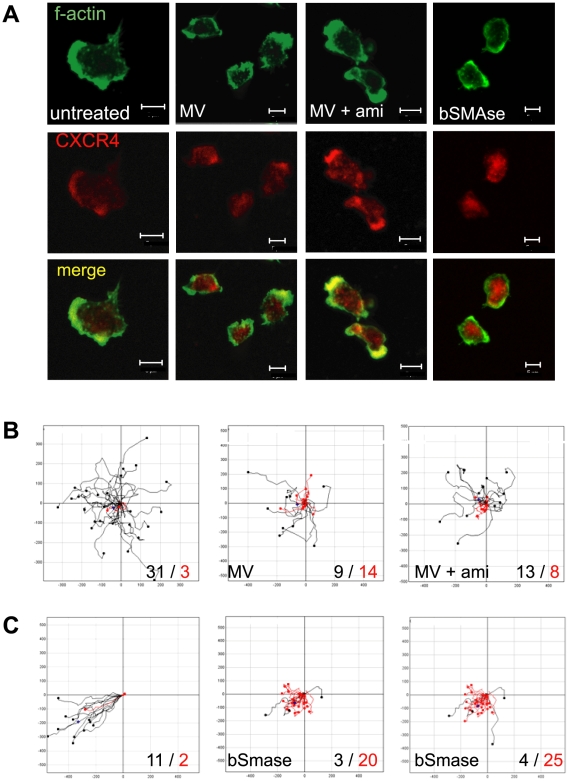
Ceramide accumulation abolishes redistribution of CXCR4 and affects T cell chemotactic motility. (A) T cells left untreated (first column) or exposed to MV (second column) alone or after a 2 h pretreatment with amitriptilyne (third column) or to bSMase (fourth column) were seeded onto FN and stained for f-actin and CXCR4. Size bars represent 5 µm. (B and C) T cells were left untreated (each: left panel) or pretreated with amitriptyline ((B), right panel) or not before exposure to MV for 2 h at 4°C ((B), middle panel) or treated with bSMase ((C), middle and right panels) for 2 hrs at 37°C and seeded onto FN with addition of SDF-1 (1,5 µg/ml). Migration of cells on FN in response to SDF-1 was recorded over a time period of 10 min and the individual velocities of cells and their trajectories were calculated using ImageJ software. Tracks of cells faster than an arbitrary velocity threshold of 7 µm/min in B, or 9 µm/min in C, are indicated in black, those slower in red in each panel. Examples shown are representative of three individual experiments including different donors with a total of at least 120 cells recorded for each experimental setup.

MV-induced ceramide generation efficiently interferes with stimulated actin cytoskeletal rearrangements as required for polarisation, and this could also impact on T cell motility. We thus analysed migration of T cells on FN in the presence of the chemokine SDF-1. Both bSMase and MV exposure abolished polarisation of T cells on FN (Fig. 3A and 4A), and thus, the SDF-1 receptor CXCR4, accumulated at the leading edge of untreated T cells (Fig. 5A, left panels), but not in bSMase or MV-treated cultures (Fig. 5A, second and fourth panels). There, CXCR4 apparently was retained in cytosolic clusters, which were even partially retained when cells were pretreated with amitriptyline prior to MV exposure (Fig. 5A, third panels) though in these cultures, a fraction of CXCR4 localised to the leading egde. When seeded onto FN alone, T cells of all cultures analysed barely migrated (not shown). Upon addition of SDF-1, T cells not exposed to MV migrated efficiently and, as revealed by the tracks recorded (exemplified in Fig. 5B) almost directionally, with 31 out of 34 exceeding an arbitrary threshold velocity of 7 µm/min. In contrast, a substantial fraction of cells remained below this velocity in MV-exposed cultures and interestingly, apparently circled rather than migrated (Fig. 5B). Indicating that these alterations related to SMase activation, pre-treatment with amitriptyline at least partially rescued T cell motility in this system (Fig. 5B), which was also effectively blocked upon exposure of T cells to bSMase (Fig. 5C). Altogether these data indicate that ceramide accumulation, as exemplified for that induced by bSMase and a model ligand, MV, efficiently ablates directed motility of T cells and presumably thereby, T cell recruitment to sites of inflammation.

## Discussion

Ceramides regulate the biophysical property of the plasma membrane, but also transform small cholesterol/SM enriched membrane microdomains enriched into platforms where receptors are concentrated and scaffolds for assembly of membrane proximal signalling complexes are provided [2], [36]–[41]. Their role in signalling initation and promotion has mainly been analysed after death receptor ligation followed by apoptosis [2],[36]. Importantly, ceramides interfere with activation of the PI3 kinase and their downstream effectors [32],[42],[43]. In addition, ASM activation has been linked to PP2A-dependent dephosphorylation of ezrin, a member of the cytoskeletal linker ERM proteins and thereby, loss of membrane protrusions in MCF-7 cells [17]. In mobile cells such as T lymphocytes where cytoskeletal reorganisations are of direct functional importance the impact of SMase activation is ill defined as yet.

T cell silencing induced by MV contact is characterised by PI3K inhibition, RhoA activation, membrane transformation in raft-like platforms, loss of membrane protrusions and ezrin phosphorylation [22] all of which are compatible with ceramide generation, and indeed, MV promoted ceramide generation and changes in cell morphology and motility resembled those seen upon exposure of cells to bSMase or C_16_-ceramide (Fig. 3, 4). Interestingly, MV induced activation of both NSM (likely representing NSM2 [44]) and ASM (Fig. 1F). In our system, activation of ASM may even rely on that of NSM, because pharmacological or genetic interference with NSM efficiently prevented extrafacial ceramide accumulation (predominantly resulting from ASM action on extrafacial SM and therefore efficiently prevented by amitrityline) both an Jurkat and primary human T cells (Fig. 1H, and not shown for Jurkat cells). NSM2 localises or can be recruited to the inner leaflet of the plasma membrane where it catalyses hydrolysis of sphingomyelin which is, however, not very abundant there [44]. Similar to that by MV, ASM activation and induction of surface ceramides by TNF-α relies on initial activation of NSM [45], yet underlying mechanisms remain unclear. Possibly, NSM-catalysed ceramide generation in the inner leaflet triggers ASM surface display by its established activity in promoting vesicle fusion [39],[46], which may include that of ASM containing membraneous compartments with the plasma membrane.

The mode of how MV activates SMases remains unresolved at present. It clearly does so by surface contact which is evident from the experimental conditions (binding in the presence of FIP) and the fast kinetic of SMase activation. As established, MV externally interacts with GM1 binding domains in primary T cells [28], and though CD150, the identified high affinity receptor required for MV entry, can localise to rafts [47], it is unlikely to substantially contribute to MV-induced SMase activation in T cells. This is because the latter occurs in resting primary T cells where CD150 expression is low [48],[49] and in Jurkat cells which do not express this protein on their surface [50]. Since we focussed on consequences of ceramide generation on T cell functions, their role in MV entry into these cells types was not specifically addressed. Unlike that of viruses entering by endocytosis such as Sindbis virus, rhinovirus and adenovirus which induce and benefit from local ceramide production [6], [51]–[53], MV entry occurs by pH-independent fusion at the plasma membrane. For HIV, which also uses this mode of entry, ceramides were found inhibitory by preventing the lateral diffusion of CD4. Rather, inceased levels of membrane sphingolipids induced by fenretinide, shifted HIV uptake into the endolysosomal/degradative pathway [8]–[10]. If this also applies to MV remains to be determined.

MV interference with TCR- or IL-2R driven PI3/Akt kinase activation was found essential to the induction of T cell growth arrest [26],[27]. Ceramides can target the relay of PI3K signalling [33],[43],[54]. Unlike that by MV (Fig. 2), inhibition of CD4 T cell activation and proliferation after cholera toxin B (CTB) binding to and generating ceramides from rafts was strictly NSM–dependent and did not involve ASM [55]. Reasons for this discrepancy are unclear at present. Notably, NSM activation by cholera toxin only peaked much later than that induced by (after 25 min, rather than 5 min) (Fig. 1F). Moreover, MV but not CTB caused ASM activation, and thus, though not directly analysed, ceramide accumulation measured in extracts of CTB exposed cells, may not be predominantly displayed at the cell surface [55]. Further suggesting that signalling pathways modulated by CTB ligation of GM1 and MV, though both causing ceramide accumulation, may substantially differ, CTB, but not MV exposure interfered with stimulated expression of early T cell activation markers CD25 and CD69 [26],[55],[56].

Our study now provides direct evidence that ceramide generation induced in a ligand (MV) dependent or independent manner profoundly affects regulated dynamics of the T cell cytoskeleton thereby modulating the ability of T cells to interact with substrates (including the ECM), retain organised protrusions and to migrate in response to chemotactic cues (Fig. 3–5). In human T cells, exogenous ceramides efficiently interfered with particularly p-ezrin levels (Fig. 4B), which is in line with findings obtained in MCF-7 cells where these were also lost after bSMase or C_16_ exposure [17]. In MCF-7 cells, the only pERM species detected by the antibody used is p-ezrin, and thus, reduced pERM levels in response to ceramide increase detected in lysates corresponded to those in immunofluorescence [17]. In T cells, moesin is much more abundant than ezrin, and interestingly, levels of p-moesin were much less affected by bSMase or C_16_ (Fig. 4B). Ezrin and moesin are important in T cell activation as directly documented after targeted disruption of both proteins [35]. Ablation of ezrin alone did not affect partitioning of talin, PKC-θ or p-ZAP-70 to the IS or CD43, p85 or ezrin binding protein 50 (EBP50) into the distal pole complex, and this was also observed when moesin was additionally silenced by RNAi. However, moesin was incompletely silenced and thus functionally compensated in partitioning membrane proteins to their respective cellular poles [35],[57]. In view of these findings, it is highly interesting that p-moesin, though largely retained and most likely representing the major pERM species detected by IF even upon ceramide increase in T cells (Fig. 4B and C) cannot functionally compensate for loss of p-ezrin in assisting αCD3/CD28-stimulated spread or FN-induced polarisation (Fig. 4A and C). Thus, aberrant sorting of otherwise preferentially p-ezrin associated proteins by p-moesin may occur as well or rather mark direct impacts of ceramides on actin polymerisation and/or branching in T cells.

As another direct consequence of ceramide-dependent interference with cytoskeletal dynamics, responsiveness to migrational cues is impaired (Fig. 5). Underlying mechanisms are likely to include the inability of MV or bSMase exposed cells to polarise on FN and thus, to redistribute CXCR4 to a leading edge and to enable directed migration (Fig. 5A). Though this was not directly assessed in our system where SDF-1 was directly added to the culture medium, the more circulating movement of ceramide-enriched T cells may reflect the more general than polarised signal perception by CXCR4 which per se is not known to be affected upon MV exposure (Fig. 5B and C). The general interference of ceramide generation with actin cyoskeletal rearrangement is, however, likely to be directly responsible for the block in T cell motility observed in our system which may even be more pronounced when analysed in a 3D environment, where leukocyte motility is almost entirely dependent on actin flow [58],[59]. It will be interesting to see as to whether in these systems, which are currently adopted, the obviously less effective signalling by MV may equal to that seen after bSMase exposure (Fig. 5B and C).

Though also not experimentally addressed as yet, ceramide generation, particularly when induced at a focal basis could be predicted to affect formation of cellular interfaces such as the IS and thereby directly impact on the efficiency of T cell conjugation and activation. In support of this notion, MV signalling shown to induce ceramides on T cells (Fig. 1), has been found to contribute to destabilisation of conjugates formed between infected DCs and T cells [60]. To what extent ceramide generation on T cells impacts on infection-dependent immunomodulation by pathogens is largely unknown. Experimental infections performed in AsmKO mice so far involved pathogens uptake of which is ASM dependent, and thus have been evaluated on differences relating to susceptibility to infection. Though Asm per se enhances Sindbis virus uptake, it also accounts for SV induced cell apoptosis and, as documented more recently, production of highly virulent particles, thereby at least partially explaining the enhanced sensitivity to CNS infection and virus production in AsmKO mice [51],[52]. Nsm2/3KO mice have not yet been experimentally infected, and in common with AsmKO mice not systematically analysed with regard to the impact of SMases on development and function of T cells which, according to our in vitro findings, could be a highly interesting avenue to follow.

## Materials and Methods

### Ethics statement

Primary human cells were obtained from the Department of Transfusion Medicine, University of Würzburg, and analysed anonymized. All experiments involving human material were conducted according to the principles expressed in the Declaration of Helsinki and ethically approved by the Ethical Committee of the Medical Faculty of the University of Würzburg.

### Cells, virus and infection experiments

Primary human T cells were enriched from peripheral blood obtained from healthy donors by Ficoll gradient centrifugation followed on nylon wool columns and, as Jurkat cells, maintained in RPMI1640/10% FCS. Spleen cells were isolated from Asm–/– knockout mice (AsmKO) and their C57BL6 wild-type littermates. MV wild-type strain WTF and the MV recombinant MGV (both grown on human lymphoblastoid BJAB cells in RPMI1640/10% FCS) were titrated on marmoset lymphoblastoid B95a cells (kept in RPMI1640/10%FCS). For exposure experiments, MV grown on BJAB cells was purified by sucrose gradient ultracentrifugation as was the MOCK control from uninfected BJAB cells. T cells were cocultured with MV or, when indicated MGV (at a multiplicity of infection (m.o.i.) of 1 each) or a MOCK preparation (used at a corresponding protein/volume concentration) in the presence of a fusion inhibitory peptide Z-D-Phe-L-Phe-Gly-OH (Bachem, Heidelberg, Germany; 200 µM in DMSO) to prevent infection of T cells throughout the experiment for 2 hrs at 4°C.

### Ceramide/SMase detection

For surface detection of ceramides and ASM we adopted an assay previously described [34]. Briefly, cells (each 3×10^6^) stimulated for the time intervals indicated (α-CD28 (clone CD28.2; 1 µg/ml; Beckton-Dickinson Biosciences Pharmingen, Heidelberg, Germany), MOCK, MGV or MV) were fixed in 1% formaldehyde and incubated with α-ceramide IgM (clone MID 15B4, Alexis) or polyclonal rabbit α-ASM IgG (H-181; Santa Cruz) over night at 4°C and washed extensively in PBS.

Cell bound antibody was desorbed for 30 sec with ice cold 100 mM glycine-HCl, pH 3.0, and spotted onto nitrocellulose following neutralisation with 100 mM Tris-HCl pH 8.0. Antibodies were detected by goat-α-mouse IgM (Dianova, Hamburg, Germany) or IgG respectively conjugated to peroxidase (New England Biolabs, Frankfurt, Germany) detected using ECL and quantified using AIDA software (Raytest). Alternatively, cell surface ceramides were detected using FACsCALIBUR after incubation with specific primary and Alexa-488-conjugated secondary α-mouse IgM (MolecularProbes).

ASM activity was determined as previously described [61] using T cell extracts, while membrane enriched fractions were used for detection of NSM activity [62] with 0,01 µCi N-methyl-^14^C-sphingomyelin used as a substrate at 37°C for 1 hr at pH 5.0 for ASM and pH 7.4 for NSM in the presence of Mg2+. ^14^C-phosphocholine produced was extracted by chloroform∶methanol (2∶1, vol/vol) and measured by liquid scintillation counting of the upper phases. Values indicated represent means of at least three independent experiments involving different donors.

### siRNA tranfection, RT-PCR analysis and Western Blot analysis

For silencing of NSM2, human T cells were nucleofected twice with a two days interval according to the manufacturer's protocol (Amaxa) with siRNA targeting human *SMPD3* (NSM2) specific [63] or, for control, a scrambled siRNA (Eurogentec, Belgium). Before cells were recruited into the respective experiments (after 96 hrs), aliquots were harvested for nucleic acid extraction (Qiagen, RNAeasy Kit) and subsequent RT-PCR analyses. Forward 5′-GCCCTTATCTTTCCATGCTACTG-3′ and reverse 5′-ACAGAGGCTGTCCTCTTAATGCT-3′ primers were used for specific *SMPD3* amplification. Alternatively, protein extracts prepared from transfected cells were used for detection of NSM2 by immunoblot analysis using a NSM-specific goat polyclonal antibody (Clone C-13, Santa Cruz). p-ERM (Cell Signalling, Frankfurt, Germany) or moesin (clone 38/87 produced in our laboratory)-specific and secondary HRP-conjugated goat α-rabbit (Cell Signalling, Frankfurt, Germany) or goat α-mouse (Dianova, Hamburg, Germany) antibodies were used to detect the corresponding proteins by WB analysis. Signals obtained after ECL development were acquired and quantified using the AIDA software program (Raytest, Straubenhardt, Germany).

### Immunostaining and scanning electron microscopy (SEM)

T cells (when indicated pre-exposed for 2 hrs to amitriptyline (10 µM), GW4869 (1,3 µM), recombinant bacterial sphingomyelinase (12,5 mU/ml, all Sigma-Aldrich, Taufkirchen, Germany) or 50 µM C_16_- (BIOMOL, Hamburg, Germany) or dh-C_16_-ceramide (Avanti Polar Lipids, Otto Norwald GmbH, Germany)) were transferred onto 8-chamber slides (LabTekII, Nunc, Wiesbaden, Germany) precoated with α-mouse IgG (Dianova, Hamburg, Germany) (2 hrs at 37°C) and subsequently activated by CD3- (clone UCHT-1) and CD28-specific antibodies (clone CD28.2)(each 1 µg/ml)(both Beckton-Dickinson Biosciences Pharmingen, Heidelberg, Germany) for 10 min at 37°C. Alternatively, T cells were transferred for 30 min to chamber slides coated with fibronectin (FN)(20 µg/ml in PBS; Sigma, München, Germany). For immunostaining, cells were fixed in paraformaldehyde (4% in PBS) and stained for membrane ceramide (clone MID 15B4, Alexis) or, after permeabilisation (0.1% Triton X-100) for CD43, CXCR4 (clone 12G5, both: Santa Cruz) or p-ERM (Cell Signalling, Frankfurt, Germany); actin was detected using Alexa488 or −594 conjugated phalloidin (all Molecular Probes, Karlsruhe, Germany). Fluorochrome G (Southern Biotech, Eching, Germany) mounted samples were analysed by confocal laser scanning microscopy (Laser Scan Microscope, LSM510 Meta, Software version 3.2, SP2; Axiovert 200 M microscope, Objective: 63×; aperture 1.4 plan apochromat; when indicated, vertical z-stacks were acquired (20 optical planes, 0.5 mm distance) and 3D deconvolutions were performed (by using Zeiss software). For SEM, cells were seeded onto FN coated slides for 30 min at 37°C and fixed by addition of 6.25% glutaraldehyde in 50 mM phosphate buffer (pH 7.2) for 10 min at RT and subsequently at 4°C overnight. After a washing step, samples were dehydrated stepwise in acetone, critical point dried and sputtered with platin/paladium before SEM analysis (Zeiss DSM 962).

### Proliferation and migration assay

Proliferation of human T cells or murine spleen cells was determined after pre-incubation with antibodies directed against human or murine CD3/CD28 (each 1 µg/ml in PBS for 30 min at 4°C) and subsequent activation by seeding onto plate bound α-mouse IgG (coated onto 96 well plates at 10 µg/ml in 50 mM Tris pH 9.0 at 4°C over night) for 72 hrs (including a final 18 hrs pulse labeling with ^3^H thymidine (0,5 µCi/well)). When indicated, cells were pretreated with fumonisin B2 (50 µM) or myriocin (50 nM) (both Sigma-Aldrich, Germany) amitriptilyne, GW4869 or siRNA transfection as detailed above).

For tracking experiments, 5×10^4^ T cells (pre-treated or not as indicated) were resuspended in 0,5%BSA/PBS supplemented with 1,5 µg/ml SDF-1 and placed onto FN-coated slides (μ-SlideVI; Ibidi). Before use slides were coated with 20 µg/ml FN in PBS at 4°C overnight, washed three times with PBS and blocked for 1 h at 37°C with 2,5%BSA/PBS. Cell images were scanned with a Laser Scanning system (LSM 510 Meta, Zeiss) based on an inverted microscope (Axiovert 200 M, Zeiss) through an objective (40×, oil, NA = 1,3 Plan-Neofluar) at 37°C. An Argon/2 (488 nm) laser was used for differential interference contrast (DIC). Images were acquired every 30 sec for 10 min, trajectories and mean velocity of individual cells was determined using ImageJ software (NIH; http://rsb.info.nih.gov/ij/).
